# Health of Soldiers and Clergymen

**Published:** 1879-01

**Authors:** 


### HEALTH OF SOLDIERS AND CLERGYMEN.

The British nation has ordered a commission to determine the best means of securing the health of soldiers, in all situations and in every particular; and scientific men have been dispatched to different countries, for the purpose of collecting information on the subject. The English have found that it costs too much to fit a soldier for his place, unless he can live for some years afterwards. If it is of national importance to secure the health of soldiers, whose office is to kill men, and if it was matter of economy with the Southern planter to study the health of his slaves, ought not these facts to suggest to the Church, as a matter of dollars and cents, if from no other motive, the propriety of devising means to preserve and promote the health of theological students, especially as the foundation next in importance to the Bible for a true orthodoxy is, a sound head and a healthy body.

